# A233 CANADIAN EXPERIENCE OF ENDOSCOPIC ULTRASOUND-GUIDED GALLBLADDER DRAINAGE: A CASE SERIES

**DOI:** 10.1093/jcag/gwae059.233

**Published:** 2025-02-10

**Authors:** S X Jiang, C Thilakanathan, R Winter, M F Byrne, A Jain, D Motomura, S Gan, R Trasolini

**Affiliations:** The University of British Columbia Faculty of Medicine, Vancouver, BC, Canada; The University of British Columbia Faculty of Medicine, Vancouver, BC, Canada; The University of British Columbia Faculty of Medicine, Vancouver, BC, Canada; Vancouver General Hospital, Division of Gastroenterology, University of British Columbia, Vancouver, BC, Canada; Vancouver General Hospital, Division of Gastroenterology, University of British Columbia, Vancouver, BC, Canada; Vancouver General Hospital, Division of Gastroenterology, University of British Columbia, Vancouver, BC, Canada; Vancouver General Hospital, Division of Gastroenterology, University of British Columbia, Vancouver, BC, Canada; Vancouver General Hospital, Division of Gastroenterology, University of British Columbia, Vancouver, BC, Canada

## Abstract

**Background:**

Endoscopic ultrasound-guided gallbladder drainage (EUS-GBD) is an emerging treatment for acute cholecystitis in patients who are poor surgical candidates. There is a lack of EUS-GBD data in the Canadian setting, which may differ from its global counterparts in pattern of healthcare availability, aging population, and use of conscious sedation.

**Aims:**

To describe outcomes following EUS-GBD in the Canadian setting.

**Methods:**

The case series included patients undergoing EUS-GBD by an interventional gastrointestinal endoscopist in a tertiary academic centre in Canada. Data included were clinicodemographic information, procedural details, and clinical outcomes.

**Results:**

From September 2023 to September 2024, a total of 18 patients underwent EUS-GBD. Median age was 85 (IQR 75-88) years, 6 (33%) were female, and 17 (94%) were American Society of Anesthesiologists Physical Status (ASA) classification III. Indication included 15 cases of acute calculous cholecystitis and 3 (17%) cases of salvage treatment for malignant biliary obstruction without alternative access. Placement was transduodenal in 10 (56%) and transgastric in 8 (44%). A coaxial double pigtail was placed in 15 cases (83%). Conscious sedation was used for 15 cases (83%), with 1 case using deep sedation with propofol and 2 with general anesthesia. All cases were technically successful followed by improvement of symptoms and biochemistry. There were no intra-procedural complications, but 3 post-procedural complications were noted. One patient had recurrent cholecystitis after 1 month due to stent occlusion despite coaxial pigtail placement, which was managed endoscopically with symptom resolution. Another patient had a buried stent, which was managed with placement of a second lumen-apposing metal stent within the first stent. A third patient had pain post-procedure suspected due to stent expansion, which self-resolved within 2 weeks. No rescue percutaneous or surgical interventions were required following EUS-GBD. No patients underwent subsequent cholecystectomy. Four patients died from underlying systemic disease, including 3 patients who underwent EUS-GBD for malignant obstruction with no recurrent cholecystitis up to time of death at 15-150 days post-procedure.

**Conclusions:**

In an elderly population and using primarily conscious sedation, EUS-GBD was safe and effective in patients with high surgical risk, underscoring the suitability of this technique for a Canadian setting.

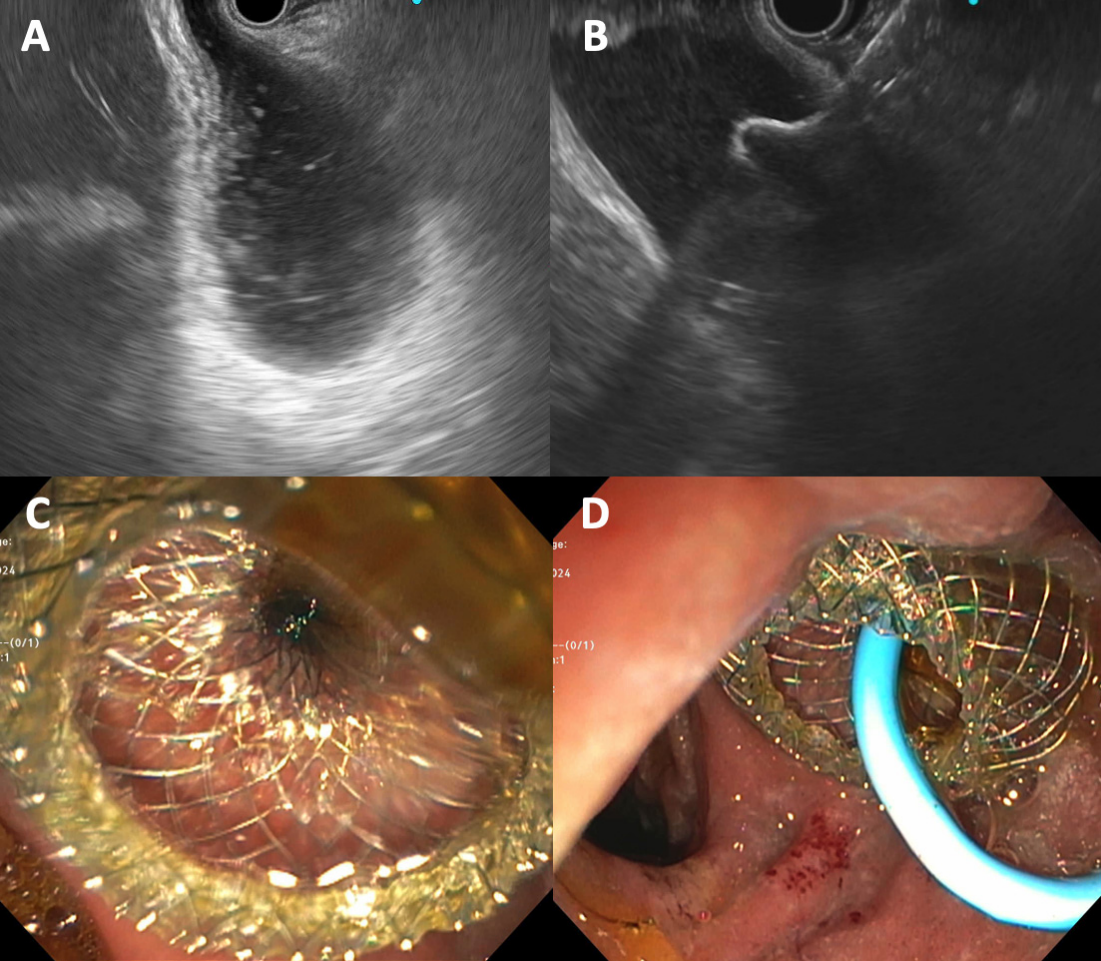

A. Gallbladder with debris/sludge on EUS. B. Distal flange of lumen apposing metal stent (LAMS) deployed within gallbladder lumen. C. Proximal flange deployed in duodenum. D. Coaxial double pigtail placed within LAMS.

**Funding Agencies:**

None

